# Lack of association between serological evidence of past *Coxiella burnetii *infection and incident ischaemic heart disease: nested case-control study

**DOI:** 10.1186/1471-2334-5-61

**Published:** 2005-07-20

**Authors:** Conall McCaughey, Liam J Murray, James P McKenna, Peter V Coyle, Hugh J O'Neill, Dorothy E Wyatt, Jayne V Woodside, John WG Yarnell, Pierre Ducimetiere, Annie Bingham, Philippe Amouyel, Michele Montaye, Dominique Arveiler, Bernadette Haas, Jean Ferrieres, Jean-Bernard Ruidavets

**Affiliations:** 1 Regional Virus Laboratory, Royal Hospitals, Grosvenor Road, Belfast BT12 6BA, UK; 2 Department of Epidemiology and Public Health, Mulhouse Building, Queen's University of Belfast, BT12 6BJ, UK; 3 Department of Medicine, Mulhouse Building, Queen's University of Belfast BT12 6BJ, UK; 4 INSERM U258, Epidemiologie cardio-vasculaire et metabolique, Hopital Paul Brousse, 94807 Villejuif Cedex, France; 5 INSERM U508, Institut Pasteur de Lille, Lille, France; 6 Laboratoire d'Epidémiologie et de Santé Publique, Strasbourg, France; 7 INSERM U558, Faculté de Médicine Purpan, Toulouse, France

## Abstract

**Background:**

Coxiella burnetii causes the common worldwide zoonotic infection, Q fever. It has been previously suggested that patients who had recovered from acute Q fever (whether symptomatic or otherwise) may be at increased risk of ischaemic heart disease. We undertook this study to determine if past infection with *Coxiella burnetii*, the aetiological agent of Q fever, is a risk factor for the subsequent development of ischaemic heart disease.

**Methods:**

A nested case-control study within the *Prospective Epidemiological Study of Myocardial Infarction *(PRIME). The PRIME study is a cohort study of 10,593 middle-aged men undertaken in France and Northern Ireland in the 1990s. A total of 335 incident cases of ischaemic heart disease (IHD) were identified and each case was matched to 2 IHD free controls. Q fever seropositivity was determined using a commercial IgG ELISA method.

**Results:**

Seroprevalence of Q fever in the controls from Northern Ireland and France were 7.8% and 9.0% respectively. No association was seen between seropositivity and age, smoking, lipid levels, or inflammatory markers. The unadjusted odds ratio (95% CI) for Q fever seropositivity in cases compared to controls was 0.95 (0.59, 1.57). The relationship was substantially unaltered following adjustment for cardiovascular risk factors and potential confounders.

**Conclusion:**

Serological evidence of past infection with *C. burnetii *was not found to be associated with an increased risk of IHD.

## Background

Q fever is a globally distributed, common, zoonotic infection caused by the bacteria *Coxiella burnetii*. A large proportion of cases of *C. burnetii *infection are asymptomatic. Where symptomatic infection occurs, typical signs and associated symptoms are headache, pyrexia, and respiratory tract infection including atypical pneumonia. Hepatitis may also occur. Chronic infection is well recognised, usually in the form of Q fever endocarditis.

Various seroepidemiological and molecular biology approaches have suggested a potential role of various viral and bacterial infections in the development of atherosclerosis. In this context it has been previously suggested that patients who recover from acute Q fever (whether symptomatic or otherwise) may be at increased risk of ischaemic heart disease(IHD)[1,2]. The first of these studies was a retrospective case-control study, a study design that is subject to several important biases including difficulty in ascertaining the temporality of relationships, and the second has been criticised for failing to adjust for important confounders[3]. Until now no prospective studies have examined this issue. We present a prospective investigation, examining the relationship between *C. burnetii *seropositivity and incident cardiovascular disease in a large cohort study of middle aged men.

## Methods

### Study design

The study was a nested case-control study within the Prospective Epidemiological Study of Myocardial Infarction (PRIME) study, which is a cohort study of middle-aged men in France and Northern Ireland (Belfast). The original purpose of this study was to investigate the relative roles of various risk factors on the development of ischaemic heart disease. Recruitment and examination methods have been fully described previously [4,5] but are briefly summarised here. A total of 10,593 men aged between 50–59 years were recruited from industry, various employment groups and general practices in Lille, Strasbourg, Toulouse and Belfast between 1991 and 1993. The sample was recruited to broadly match the social class structure of the background population. Each subject completed self-administered questionnaires on demographic, socio-economic factors and dietary habits after informed consent was obtained. Their responses were checked by medical staff and additional data collected during clinic attendance on educational level, occupational activity, personal and family history, tobacco and alcohol consumption, and physical activity. The London School of Hygiene and Tropical Medicine Cardiovascular (Rose) Questionnaire for Chest Pain on Effort and Possible Infarction [6] was also administered.

### Clinical examination

Baseline investigations included a standard 12-lead electrocardiogram and standardised blood pressure measurements (measured on 2 occasions in the sitting position) using an automatic sphygmomanometer (Spengler SP9). Anthropometric measurements included height and weight without shoes and waist and hip circumferences. Subjects were considered to have a history of IHD at entry if they had one of the following: myocardial infarction (MI) and/or angina pectoris diagnosed by a physician, electrocardiographic evidence of MI, or a positive answer to the Rose questionnaire. There were 9,758 subjects without a history of IHD at entry into the study.

### Case-control selection and follow-up

Subjects were contacted annually by letter and asked to complete a clinical event questionnaire. Phone contact was established with non-responders or their general practitioner. Coronary cases were defined as the presence of at least one of the following: non-fatal MI, death from IHD, or angina pectoris. Five-year follow-up has been completed (98% follow-up was achieved). The total number of incident IHD cases identified was 335. Each case was matched to 2 controls, who were study participants of the same age (± 3 years) recruited in the same centre on the same day (± 2 days) as the corresponding case and were free of IHD on the date of the ischaemic event of the case.

### Laboratory methods

Baseline venous blood samples were collected at the start of the study after a 12 hour fast and centrifuged within 4 hours. All assays were performed blind to the investigating centre and by consecutive number so that samples from all 4 centres were included in each analytical run. All procedures were standardised between centres. Among other measurements, total and HDL cholesterol, triglycerides, and fibrinogen were assayed at recruitment on all subjects. C-reactive protein (CRP) and interleukin 6 (IL-6) were assayed on stored samples from cases and controls. Enzymatic methods were employed to measure total cholesterol and triglycerides using a commercial kit in an automatic analyser (Boehringer Mannheim, Germany). HDL cholesterol was measured by an enzymatic method (Boehringer Mannheim, Germany) after precipitation using phosphotungstate magnesium chloride. LDL cholesterol was calculated according to Friedewald and colleagues [7] Fibrinogen was measured according to the method of Clauss[8] High sensitivity CRP was measured by immunonephelometry (hs-CRP: Dade Behring, Reuil-Malmaison, France). IL-6 was measured by ELISA (R&D, Europe).

### Q fever ELISA testing

The presence of IgG antibodies to Phase II *C. burnetii *in the baseline serum specimen was performed using a commercially available indirect ELISA test kit (Vircell, Granada, Spain. Cat. No. G1001). The standard manufacturer's protocol was followed for testing, use of controls, and interpretation The antigen used in the Vircell IgG assay is *C. burnetii *phase II Nine Mile, grown in cell culture. The cut off for a positive test result was based on the manufacturers stipulated guidelines. Provided that Optical Densities (OD) for positive, negative and cut off controls fell within the manufacturers stated guidelines the test was considered valid. The mean OD for cut off serum was determined. An antibody index was then determined for each sample tested Antibody Index = (sample OD/cut off serum mean OD) × 10. The results were then interpreted in line with the manufacturer's guidance as follows: <9 Negative; 9–11 Equivocal; >11 Positive.

### Statistical methods

Smoking status was categorised as never smoked, ex-smoker, or current smoker of <20 or = 20 cigarettes a day. Alcohol intake was calculated in grams per week and categorised into none, <225 g/week and = 225 g/week. The following skewed variables were log transformed before inclusion in analyses: triglycerides, CRP, and IL-6. Equivocal Q fever results were treated as seropositive (n = 3). In the controls, Chi-square tests and t-tests were used to examine the differences in prevalence of Q fever seropositivity between Northern Irish and French subjects, the relationship between seropositivity and age and smoking status, and between seropositivity and lipid levels and markers of inflammation (fibrinogen, CRP and IL-6). Conditional (matched) logistic regression analysis was used to examine the relationship between Q fever seropositivity and case-control status. The relationship was adjusted for age, smoking, body mass index, systolic and diastolic blood pressure, social class, physical activity, and alcohol intake at baseline. Further adjustment was undertaken for lipids (total cholesterol, HDL cholesterol and triglycerides) and then for markers of inflammation. Finally, a country/Q fever seropositivity interaction term was entered into the model. All analyses were performed using Stata version 8.

Ethical approval was obtained from the Queen's University of Belfast Ethical Committee

## Results

Serum was available for Q fever testing from 320 (95.5%) of the 335 cases and 622 (92.8%) of 670 controls. Q fever results were available for the case and at least one control in 317 cases (94.6%). Among the controls, there was no difference in the seroprevalence of Q fever antibodies between Northern Ireland and France; 7.8% and 9.0% seropositive respectively. Within France, 12.4% of controls from Toulouse were seropositive, while seropositivity in the other 2 centres was 7.0% (χ^2 ^= 2.99, df 1, p = 0.08). There was no difference in age between seropositives and seronegatives; mean age (SD) was 54.9 years (3.11) and 55.2 years (2.69) respectively. No association was seen between seropositivity and age, smoking, lipid levels, or inflammatory markers (Table 1).

**Table 1:** Relationships between seropositivity for Q fever and characteristics of control subjects

		Mean (SD)		p-value
		Seronegative	Seropositive	

Age (years)		55.2 (2.69)	54.9 (3.11)	0.45
Total cholesterol		2.23 (0.42)	2.15 (0.31)	0.20
HDL		0.48 (0.12)	0.48 (0.15)	0.69
Triglycerides*		1.37 (1.72)	1.35 (1.68)	0.81
Fibrinogen		3.37 (0.98)	3.53 (1.10)	0.16
CRP*		1.39 (2.73)	1.32 (2.01)	0.76
IL-6*		1.53 (1.97)	1.39 (2.73)	0.14
				
		Number (%)	Number (%)	

Smoking	never smoked	223 (25.8)	14 (18.2)	
	ex-smoker	360 (41.6)	38 (49.4)	
	current smoker < 20/day	164 (18.9)	11 (14.3)	
	current smoker ≥ 20/day	118 (13.6)	14 (18.2)	0.21

* geometric mean

The unadjusted and adjusted relationships between Q fever seropositivity and case-control status are shown in Table 2. The odds of Q fever seropositivity in cases compared to controls was not elevated, either before or after adjustment for the baseline variables age, smoking, body mass index, systolic and diastolic blood pressure, social class, physical activity, and alcohol intake. Further adjustment for lipids also had little effect on observed odds ratios. The relationship strengthened slightly on adjusting for the inflammatory markers fibrinogen, CRP and IL-6, but was not statistically significant. A country/Q fever seropositivity interaction term was not significant when added to the fully adjusted model.

**Table 2:** Unadjusted and adjusted relationships between Q fever seropositivity and case-control status.

	Unadjusted odds ratio (95% CI)	Adjusted odds ratio^1 ^(95% CI)	Adjusted odds ratio^2 ^(95% CI)	Adjusted odds ratio^3 ^(95% CI)
Q fever status				
Seronegative	1.00 (reference category)	1.00 (reference category)	1.00 (reference category)	1.00 (reference category)
Seropositive	0.95 (0.57 1.57)	0.96 (0.56, 1.64)	1.04 (0.59, 1.82)	1.20 (0.66, 2.18)

^1 ^adjusted for age, smoking, body mass index, systolic and diastolic blood pressure, social class, physical activity, and alcohol intake

^2 ^further adjustment for total cholesterol, HDL cholesterol, and triglycerides

^3 ^further adjustment for fibrinogen, CRP, and IL-6

## Discussion

Atheroma is an inflammatory condition which has been putatively associated with infections including a range of bacterial infections. Against this background it has been suggested that the long-term sequelae of *C. burnetii *infection may include an elevated risk of cardiovascular disease[1,2]. Original tentative suggestions of a link were in the form of a case report of acute MI in a patient with Q fever infection[9]. More recently Enders et al. have suggested that *C. burnetii *infection may have a modest association with coronary artery disease. This is based on the evaluation of 155 consecutive patients undergoing coronary angiography using *C. burnetii *serology[1] Further positive associations have been reported in a follow-up study of people infected in 1983 in a large outbreak of Q fever in Switzerland[10] However this study was criticised for not controlling for current and past cigarette smoking as a potential explanation for the excess cardiovascular morbidity and mortality observed[3]. In this nested case-control study we were able to adjust for smoking and a range of other potential confounders, but this adjustment had little effect on observed odds ratios. We did not find any association between serological evidence of prior Q-fever infection and incident IHD. However, it is possible that selection/survivor bias may have occurred to some extent in the study. People with more severe Q fever infections, who may be more likely to develop some arterial disease if an association exists, may not have been enrolled in the study because of illness or death. In the Swiss study acute Q fever was found to be associated more closely with stroke than with myocardial infartion, however the stroke outcome could not be tested in our study. The reported chronic fatigue syndrome associated with Q fever appears not to be associated with cardiac disease. [11]

### Smoking

Previous literature has suggested an association between smoking status and Q fever. In a large outbreak of Q fever pneumonia in Birmingham, UK in 1989, 60 (55%) of 110 patients for whom smoking data were available were current smokers, 28 (25%) were ex-smokers, and only 22 (20%) had never smoked[3]. A subsequent case-control study in the same cohort demonstrated smoking to be a risk factor for Q fever[12] Smoking was also shown to be an independent risk factor for acquisition of Q fever infection in goat workers in rural Newfoundland[13] Several possible explanations exist for these findings. Smoking may reflect the extent of hand to mouth contact which may be important in transmission of the infection, especially in an occupational exposure setting. Alternatively, smokers may be more likely to get symptomatic rather than asymptomatic disease. Our finding of no association between smoking status and seroprevalence would tend to favour the second explanation, assuming that the reported association with smoking and symptomatic Q fever is valid. Alternatively, an explanation of our finding is that that there is no increased risk of infection, either silent or clinical, in smokers.

### Geographic differences

Differences in seroprevalence between Northern Ireland and France might have been expected, as they are very different in terms of topography and farming practice. However, the cohort recruitment methods employed in both countries were likely to exclude farmers. There was some evidence of higher prevalence in the southern French centre compared to the other centres, although conventional statistical significance was not achieved. An overall seroprevalence figure of almost 10% in populations not known to be occupationally exposed to Q fever testifies to the ubiquity of infection with this zoonotic agent in human populations in Europe.

## Conclusion

This prospective study did not find any association between serological evidence of past infection with *C. burnetii *and IHD.

## Competing interests

The author(s) declare that they have no competing interests.

## Authors' contributions

CMcC and LJM jointly conceived the study, secured funding, participated in its design and coordination and co-drafted the manuscript. LJM also performed the main data analysis. JPMcK performed the Q fever serology testing and participated in the coordination of the study. HJON and PVC participated in the design and coordination of the study. DEW assisted in the coordination of the study and assisted in the editing of the manuscript. JWGY, JVW, PD, AB, PA, MM, DA, BH, JF and J-BR were responsible for the conception, design and coordination of the PRIME study in France and Northern Ireland. All authors helped revise and subsequently approved the final manuscript. CMcC and LJM are joint guarantors.

## Pre-publication history

The pre-publication history for this paper can be accessed here:


